# Infrageneric Plastid Genomes of *Cotoneaster* (Rosaceae): Implications for the Plastome Evolution and Origin of *C. wilsonii* on Ulleung Island

**DOI:** 10.3390/genes13050728

**Published:** 2022-04-21

**Authors:** JiYoung Yang, Seon-Hee Kim, Jae-Hong Pak, Seung-Chul Kim

**Affiliations:** 1 Research Institute for Dok-do and Ulleung-do Island, Kyungpook National University, 80 Daehak-ro, Buk-gu, Daegu 41566, Korea; jyyangson@knu.ac.kr; 2 Department of Biological Sciences, Sungkyunkwan University, 2066 Seobu-ro, Suwon 16419, Korea; desfilles@naver.com

**Keywords:** *Cotoneaster*, Rosaceae, eastern Asia, plastome, positive selection

## Abstract

*Cotoneaster* is a taxonomically and ornamentally important genus in the family Rosaceae; however, phylogenetic relationships among its species are complicated owing to insufficient morphological diagnostic characteristics and hybridization associated with polyploidy and apomixis. In this study, we sequenced the complete plastomes of seven *Cotoneaster* species (*C. dielsianus*, *C. hebephyllus*, *C. integerrimus*, *C. mongolicus*, *C. multiflorus*, *C. submultiflorus*, and *C. tenuipes*) and included the available complete plastomes in a phylogenetic analysis to determine the origin of *C. wilsonii*, which is endemic to Ulleung Island, Korea. Furthermore, based on 15 representative lineages within the genus, we carried out the first comparative analysis of *Cotoneaster* plastid genomes to gain an insight into their molecular evolution. The plastomes were highly conserved, with sizes ranging from 159,595 bp (*C. tenuipes*) to 160,016 bp (*C. hebephyllus*), and had a GC content of 36.6%. The frequency of codon usage showed similar patterns among the 15 *Cotoneaster* species, and 24 of the 35 protein-coding genes were predicted to undergo RNA editing. Eight of the 76 common protein-coding genes, including *ccsA*, *matK*, *ndhD*, *ndhF*, *ndhK*, *petA*, *rbcL*, and *rpl16*, were positively selected, implying their potential roles in adaptation and speciation. Of the 35 protein-coding genes, 24 genes (15 photosynthesis-related, seven self-replications, and three others) were found to harbor RNA editing sites. Furthermore, several mutation hotspots were identified, including *trnG*-UCC/*trnR*-UCU/*atpA* and *trnT*-UGU/*trnL*-UAA. Maximum likelihood analysis based on 57 representative plastomes of *Cotoneaster* and two *Heteromeles* plastomes as outgroups revealed two major lineages within the genus, which roughly correspond to two subgenera, *Chaenopetalum* and *Cotoneaster*. The Ulleung Island endemic, *C. wilsonii*, shared its most recent common ancestor with two species, *C. schantungensis* and *C. zabelii*, suggesting its potential origin from geographically close members of the subgenus *Cotoneaster*, section *Integerrimi*.

## 1. Introduction

The genus *Cotoneaster* Medik. is one of the most taxonomically challenging lineages in the family Rosaceae because of apomixes, hybridization, polyploidy, and unclear species circumscription [1,2,3,4]. The genus, comprising approximately 150 species, is mainly distributed in the northern hemisphere excluding Japan, and the most important center of diversity is the Himalayas and two neighboring southwestern provinces of China, Yunnan and Sichuan [1,2,5,6,7,8]. Owing to many complex species groups, insufficient diagnostic morphological features, and complex evolutionary processes, the infrageneric classification of *Cotoneaster* has been debated over the past 130 years. Koehne [9] recognized two subgenera primarily based on the petal characteristics: subgenus *Chaenopetalum* (spreading or rarely semi-spreading petals that are pink in the bud and white when open, and subgenus *Cotoneaster* (erect or suberect petals that are white, red, pink, or green). Yü et al. argued that the number of flowers per inflorescence is the main characteristic for infrageneric classification and proposed three sectional level infrageneric systems: *Uniflora* (1–5 flowered), *Cotoneaster* (3–15 flowered), and *Densiflos* (>20 flowers per cyme) [5]. Flinck and Hylmö acknowledged the two major groups described by Koenhe at the sectional level and further proposed a four subsection and 24-series system [9,10]. A slightly different infrageneric classification, that is, two subgenera, five sections, and 39 series, has also been proposed [11]. Two subgenera, nine sections, and 37 series (or two subgenera, 11 sections, and 35 series more accurately) have been proposed as the most recent infrageneric classification [1].

Several attempts at either restricted or broader scales have been made to evaluate previous classification systems and to hypothesize interspecific relationships within *Cotoneaster* [2,4,12,13,14]. Although phylogenies based on nuclear internal transcribed spacer (nrITS) and chloroplast non-coding region sequences provide limited resolution, Li et al. confirmed two major groups within the genus and revealed non-monophyly of four subsections and the series of Koehne’s infrageneric classification system [2,9]. Furthermore, given the major incongruence between nuclear and chloroplast data, hybridization was suggested to play an important role during the evolution of *Cotoneaster*. Most recently, much more comprehensive sampling and extensive molecular data (204 low-copy nuclear genes and complete plastome sequences) allowed Meng et al. to further evaluate infrageneric classification systems as well as to disentangle the complex evolutionary history of the genus [4]. This highly resolved phylogenetic framework supported two major groups within the genus (excluding species with strong conflicting signals); the cyto-nuclear discordance observed at both the species and clade levels was hypothesized to be attributed to frequent hybridization and incomplete lineage sorting [4]. In addition, this study suggested that the crown clade of *Cotoneaster* originated in the early Miocene (ca. 20 Ma) and that all extant species were evolved during the period from the middle Miocene (ca. 12 Ma) to the present.

**Figure 1:**
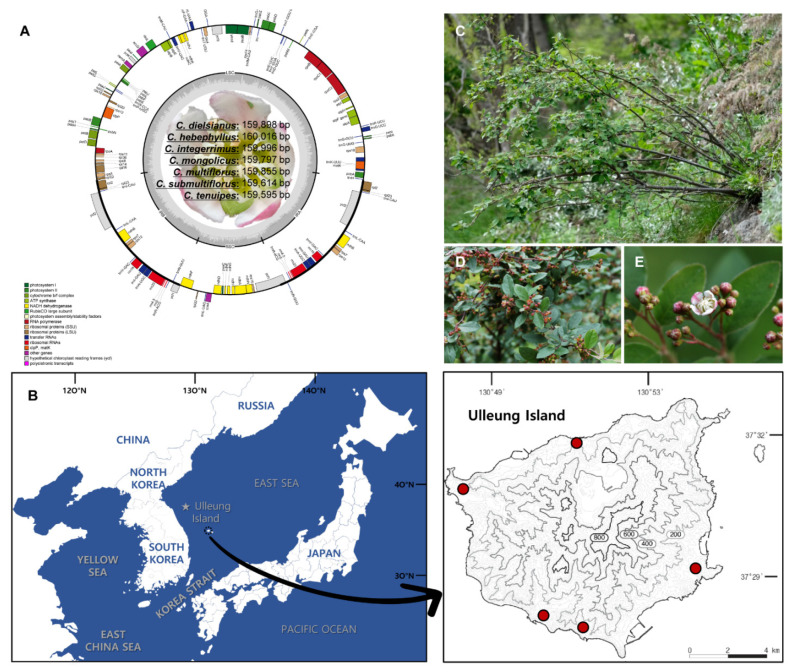
The complete plastome map of the seven newly sequenced *Cotoneaster* species in this study and *Cotoneaster wilsonii* on Ulleung Island (**A**) The genes located outside the circle are transcribed clockwise, whereas those located inside are transcribed counterclockwise. The gray bar area in the inner circle denotes the guanine-cytosine (GC) content of the genome, whereas the lighter gray area indicates the adenosine-thymine (AT) content of the genome. Large single copy, small single copy, and inverted repeats are indicated as LSC, SSC, and IR, respectively. Ψ indicates pseudogenes. (**B**) The contour map of Ulleung Island shows the population locations of critically endangered *C. wilsonii*. One typical habitat (**C**) of *C. wilsonii* on the eastern part of the island, with the inflorescence (**D**), and flowers (**E**) is shown.

The genus *Cotoneaster* is not known to occur in the mainland of South Korea and Japan [15,16], which makes the origin and evolution of *C. wilsonii* Nakai on Ulleung Island intriguing. In Korea, only two species of *Cotoneaster* are known to occur: *C. integerrimus* Medik. in North Korea and *C. wilsonii* on Ulleung Island. Ulleung Island is an oceanic volcanic island situated between the Korean Peninsula and the Japanese Archipelago; it is approximately 1.8 million years old and is home to 500 native vascular plant species. A small deciduous shrub, *C. wilsonii*, is one of more than 40 endemic species on Ulleung Island and is designated as a critically endangered species (CR B2ab(ii)) [17]. About 100 individuals of this species in five small populations on sunny cliffs at approximately 100 m above sea level are found in Ulleung Island (Figure 1). Based on its overall morphological characteristics, the Ulleung Island endemic *C. wilsonii* appears to belong to section *Cotoneaster* sensu Yü et al. [5]. Furthermore, based on its morphological and flavonoid similarities, *C. wilsonii* is suggested to be closely related to the species in sect. *Cotoneaster* series *Multiflori*, such as *C. multiflorus*, *C. submultiflorus*, *C. hebephyllus*, *C. mongolicus*, and *C. soongoricus* [14]. Of several species in the series *Multiflori*, two morphologically similar species, *C. multiflorus* and *C. hebephyllus*, have been found to have identical flavone O-glycosides to *C. wilsonii*. Furthermore, the few morphological differences and the identical flavonoid profile between *C. wilsonii* and *C. multiflorus* indicate that they are conspecific [14]. Despite its conservation status and enigmatic origin on Ulleung Island from continental parental species, there has been no attempt to conduct broad scale phylogenetic analysis of *C. wilsonii*, including that of the closely related congeneric species from the series *Multiflori* of section *Cotoneaster*.

The chloroplast genome of angiosperms usually encodes 110–130 genes with a size range of 120–160 kb and is generally recognized as a valuable genetic resource for phylogenetic and population genetic studies [18,19,20,21,22,23]. High-throughput sequencing technologies have allowed rapid accumulation of complete plastome sequences in various plant lineages, providing opportunities for comparative analyses to gain insights into plastome organization and evolution. Indeed, comparative analyses of plastomes at various taxonomic levels have revealed their basic genomic structure, gene content, gene order, and mutation hotspots, and helped improve the present understanding of intracellular gene transfer, photosynthetic evolution in parasitic plants, insular plant evolution, and plant adaptation [24,25,26,27,28,29,30,31,32,33,34,35,36]. Since the first report on the complete plastome of *Fragaria* [37] and *Malus* [38], numerous plastome sequences in the family Rosaceae have been characterized, including *Prunus* L. [39,40], *Pyrus* L. [41], *Rosa* L. [30], and *Rubus* [27,29,34,42]. Although the plastomes of *Cotoneaster wilsonii* and numerous other congeneric species have been characterized and utilized for phylogenetic analysis, respectively, no attempt has been made to understand their genomic structure, gene order, gene contents, mutation hotspots, and positively selected plastid genes within the genus [4,28].

In this study, we characterized the complete chloroplast genome sequence of seven *Cotoneaster* species (*C. dielsianus*, *C. hebephyllus*, *C. integerimus*, *C. mongolicus*, *C. multiflorus*, *C. submultiflorus*, and *C. tenuipes*) and included them as part of a broader phylogenetic framework within the genus. To gain insights into plastome organization and evolution within *Cotoneaster*, we selected the major lineages within the genus and conducted comparative analyses, including codon usage, positive selection, RNA editing sites, and mutation hotspots. We further explored the phylogenetic relationship of *C. wilsonii* relative to other congeneric species to determine its origin and evolution on Ulleung Island, Korea. Based on the plastid phylogenomic and comparative analyses, this study provides new insights into the origin and evolution of the insular endemic *C. wilsonii*, as well as the overall plastome evolution within the taxonomically challenging and horticulturally important genus, *Cotoneaster*.

## 2. Materials and Methods

### 2.1. Plant Sampling, DNA Isolation, and Plastome Sequencing, Assembly and Annotation

We sequenced a total of seven *Cotoneater* species in this study: *C. dielsianus* (13428*B; China, west Hubei), *C. hebephyllus* (68-85*A; cultivated plant originally from Sweden), *C. integerimus* (1234-82*C; cultivated plant originally from France), *C. mongolicus* (1007-86*A; China, north Guangdong), *C. multiflorus* (11334*A; China, Shaanxi), *C. submultiflorus* (208-2000*A; China, Gansu Mt. Maijii), and *C. tenuipes* (7276*A; China, West Sichuan). All but two species, *C. hebephyllus* and *C. integerimus*, are of wild origin from China. As wild materials are difficult to obtain from their geographical source areas, all plant materials were obtained from living collections (permit number of “11-2019”) at the Arnold Arboretum of Harvard University, USA (Table S1).

Fresh leaves were collected and dried using silica gel prior to DNA extraction. Total DNA was extracted using the DNeasy Plant Mini Kit (Qiagen, Carlsbad, CA, USA) and sequenced using an Illumina HiSeq 4000 (Illumina, Inc., San Diego, CA, USA), yielding a 150 bp paired-end read length, at Macrogen Co. (Seoul, Korea). The resulting paired-end reads were assembled de novo using Velvet v1.2.10 with multiple k-mers [43] with coverage ranging from 149 to 755. tRNAs were confirmed using tRNAscan-SE [44]. The sequences were annotated using Geneious R10 [45] and deposited in GenBank: *C. dielsianus* (MZ475329), *C. hebephyllus* (MZ475334), *C. integerrimus* (MZ475328), *C. mongolicus* (MZ475330), *C. multiflorus* (MZ475331), *C. submultiflorus* (MZ475332), and *C. tenuipes* MZ475333). Annotated sequence files in the GenBank format were used to draw a circular map with OGDRAW v1.2 [46].

### 2.2. Comparative Plastome Analysis

To gain insights into plastome evolution within the genus *Cotoneaster*, we selected 15 taxa and compared their genomic features using the Shuffle-LAGAN mode [47] of mVISTA [48]. The 15 taxa included seven species that we newly sequenced, Ulleung Island endemic *C. wilsonii* (NC046834), and seven species representing two major lineages within the genus: the *Chaenopetalum* group (*C. soongoricus*, *C. vandelaarii*, and *C. conspicuous*) and *Cotoneaster* group (*C. microphyllus*, *C. foveolatus*, *C. horizontalis*, and *C. franchetii*) [4]. Sequences of the 15 *Cotoneaster* plastomes were aligned using the back-translation approach with MAFFT v7.490 [49] and were manually edited using Geneious R10 [45]. Using DnaSP v6.10 [50] sliding window analysis was performed with a step size of 200 bp and window length of 800 bp to determine the nucleotide diversity (Pi) of the plastomes. Codon usage frequency was calculated using MEGA v7 [51] based on the relative synonymous codon usage (RSCU) value [52], which is a simple measure of non-uniform usage of synonymous codons in a coding sequence. The DNA code used by bacteria, archaea, prokaryotic viruses, and chloroplast proteins was used [53]. Protein-coding genes were run using the PREP suite [54] with 35 reference genes and a cut-off value of 0.8 to predict the possible RNA editing sites in 15 *Cotoneaster* plastomes. To evaluate the natural selection pressure on the protein-coding genes of 15 *Cotoneaster* plastomes, a site-specific model was developed using EasyCodeML [55] with the CODEML algorithm [56]. Seven codon substitution models (M0, M1a, M2a, M3, M7, M8, and M8a) were constructed and compared to detect the positively selected sites based on the likelihood ratio test (LRT).

### 2.3. Phylogenetic Analysis

For phylogenetic analysis, complete plastome sequences of 57 accessions of *Cotoneaster* and two accessions of outgroup *Heteromeles* were aligned using MAFFT v7.490 [49] in Geneious R10 [45]. Of *Cotoneaster* plastome sequences available in GenBank generated primarily by Meng et al. [4], we obtained the accessions with complete sequences only for the phylogenetic analysis. To determine the phylogenetic position of *C. wilsonii* on Ulleung Island, we included the plastome sequences generated by Meng et al. [4] and followed the classification system of Fryer and Hylmö [1]. These included a total of ten sections: sect. *Adpressi* (*C. perpusillus*, *C. rotundifolius*, *C. subadpressus*, *C. horizontalis*, *C. adpressus*, *C. praecox*, *C. langei*, and *C. tenuipes*), sect. *Alpigeni* (*C. sherriffli*, *C. conspicuus*, *C. astrophoros*, *C. rockii*, *C. microphyllus*, *C. cochleatus*, and *C. dammerii*), sect. *Multiflori* (*C. hebephyllus*, *C. submultiflorus*, and *C. multiflorus*), sect. *Franchetioides* (*C. dielsianus*, *C. huahongdongensis*, *C. leveillei*, *C. sternianus*, and *C. franchetii*), sect. *Rokujodaisanense* (*C. vandelaarii*), sect. *Megalocarpi* (*C. mongolicus* and *C. soongoricus*), sect. *Densiflori* (*C. serotinus*, *C. fulvidus*, *C. salicifolius*, *C. lacteus*, *C. tubinatus*, and *C. vellaeus*), sect. *Sanguinei* (*C. rubens* and *C. acuminatus*), sect. *Chaenopetalum* (*C. affinis* and *C. gamblei*), sect. *Acutifolii* (*C. foveolatus*, *C. villosulus*, *C. obscurus*, *C. moupinensis*, *C. cinerascens*, *C. acutifolius*, *C. reticulatus*, and *C. bullatus*), and sect. *Cotoneaster* (*C. zabelii*, *C. shansiensis*, *C. integerrimus*, and *C. schantungensis*). *Cotoneaster chengkangensis* was not placed in the section, whereas *C. wilsonii* was placed in subgenus *Cotoneaster*, section (and series) *Megalocarpi* sensu Fryer and Hylmö [1]. Maximum likelihood (ML) analysis based on the best-fit model of “TVM + F + R2” was conducted using IQ-TREE v1.4.2 [57]. *Heteromeles arbutifolia* was used as the outgroup, and non-parametric bootstrap analysis was performed with 1000 replicates.

## 3. Results

### 3.1. Chloroplast Genome Size and Features

The total paired-end sequence reads were *C. dielsianus* (50,739,498), *C. hebephyllus* (49,015,178), *C. integerrimus* (49,728,586), *C. mongolicus* (52,448,006), *C. multiflorus* (54,743,088), *C. submultiflorus* (42,975,682), and *C. tenuipes* (51,539,694) with coverages of 721×, 149×, 223×, 582×, 229×, 160×, and 755×, respectively (Table 1). The complete plastome length of seven *Cotoneaster* species ranged from 159,595 bp (*C. tenuipes*) to 160,016 bp (*C. hebephyllus*) (Figure 1). The large single copy (LSC) region, small single copy (SSC) region, and two inverted repeat (IR) regions ranged from 87,592 bp (*C. tenuipes*) to 87,903 bp (*C. multiflorus*), from 19,086 bp (*C. submultiflorus*) to 19,239 bp (*C. tenuipes*), and from 26,371 bp (*C. mongolicus*, *C. multiflorus*, and *C. submultiflorus*) to 26,501 bp (*C. integerrimus*), respectively (Table 1). All seven newly sequenced plastomes of *Cotoneaster* contained 131 genes, including 84 protein-coding, eight ribosomal RNA, and 37 transfer RNA genes, and their overall guanine-cytosine (GC) content was identical (36.6%) (Table 1). Furthermore, all seven plastomes contained a total of 17 duplicated genes in the IR regions, including seven tRNA, four rRNA, and six protein-coding genes. Sixteen genes (*atpF, ndhA, ndhB, petB, petD, rpl2, rpl16, rpoC1, rps12, rps16, trnA*-UGC, *trnG*-UCC, *trnI*-CAU, *trnK*-UUU, *trnL*-UAA, and *trnV*-UAC) contained a single intron, whereas *clpP* and *ycf3* each contained two introns. Of the seven plastomes of *Cotoneaster*, *C. integerrimus* contained the longest partial *ycf1* gene (1329 bp), whereas that in the others was 1206 bp long except for *C. dielsianus* (1224 bp) located in the IRb/SSC junction region. As for the complete *ycf1* gene, which is located in the IR region at the SSC/IRa junction, *C. submultiflorus* contained the shortest gene (4455 bp), whereas the others all had the same length of 5763 bp. Interestingly, all 15 plastomes of *Cotoneaster* (seven newly sequenced in this study and eight representative lineages within the genus used for comparative analysis, including *C. wilsonii*) retained the intron-containing *atpF* gene.

### 3.2. Codon Usage

**Figure 2:**
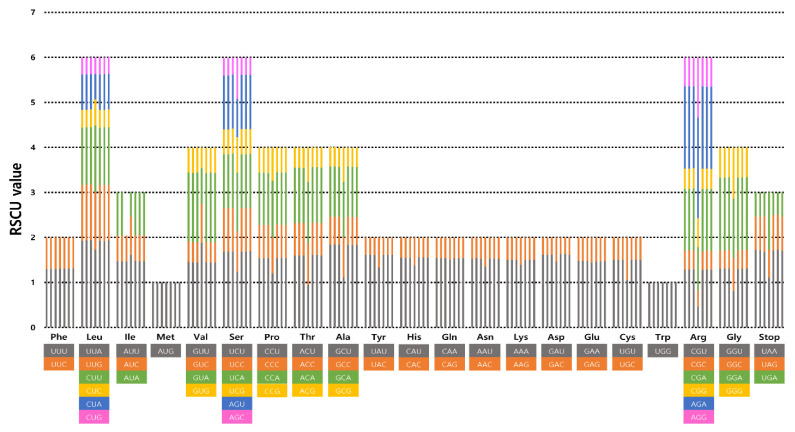
Codon distribution and relative synonymous codon usage in the plastomes of seven newly sequenced *Cotoneaster* species, Ulleung Island endemic *C. wilsonii*, and seven representative lineages within the genus. The list of species from left to right columns represent *C. dielsianus*, *C. hebephyllus*, *C. integerrimus*, *C. mongolicus*, *C. multiflorus*, *C. submultiflorus*, and *C. tenuipes*.

The frequency of codon usage in the 15 plastomes of *Cotoneaster*, representing the seven newly sequenced and eight major lineages within the genus, was calculated based on the sequences of protein-coding and tRNA genes. The results revealed that the average codon usage among the 15 species ranged from 25,868 (*C. submultiflorus*) to 26,586 (*C. horizontalis*) (Supplementary Table S2). The average codon usage for the remaining species was as follows: 26,083 for *C. conspicuous*, 26,315 for *C. dielsianus*, 26,085 for *C. foveolatus*, 26,086 for *C. franchetii*, 26,283 for *C. hebephyllus*, 26,403 for *C. integerrimus*, 26,084 for *C. microphyllus*, 26,358 for *C. mongolicus*, 26,334 for *C. multiflorus*, 26,085 for *C. soongricus*, 26,352 for *C. tenuipes*, 26,083 for *C. vandelaarii*, and 26,616 for *C. wilsonii*. The highest RSCU value was indicated in the usage of the UUA codon for leucine (1.92–1.94) followed by that of GCU for alanine (1.83–1.84) and AGA for arginine (1.82–1.84). The lowest RSCU value was indicated in the usage of AGC for serine (0.38–0.39) and GAC for aspartic acid (0.37–0.38). We found the distribution of codon types to be consistent (Figure 2), and codons AUG (M) and UGG (W) encoded methionine and tryptophan, respectively, showing no bias (RSCU = 1) (Supplementary Table S2).

The predicted number of RNA editing sites in 15 *Cotoneaster* plastomes was 63, with the same cut-off value, and 24 of 35 protein-coding genes were predicted to undergo RNA editing (Supplementary Table S3). These genes included 14 photosynthesis-related genes (*atpA*, *atpB*, *atpF*, *atpI*, *ndhA*, *ndhB*, *ndhD*, *ndhF*, *ndhG*, *petB*, *psaI*, *psbE*, *psbF*, and *psbL*), seven self-replication genes (*rpoA*, *rpoB*, *rpoC1*, *rpoC2*, *rps2*, *rps14*, and *rps16*), and three others (*accD*, *clpP*, and *matK*). We detected no RNA editing sites in 10 genes (i.e., *ccsA*, *petD*, *petG*, *petL*, *psaB*, *psbB*, *rpl2*, *rpl20*, *rpl23*, and *ycf3*), and this phenomenon was consistent among the 15 *Cotoneaster* plastomes. However, *C. microphyllus* showed RNA editing sites in one additional photosynthesis-related gene, *rps8*, making a total of 15 genes in this category. Two species, *C. franchetii* and *C. vandelaarii*, contained one additional site compared to the remaining 13 species: a total of five and nine RNA editing sites in the *ndhF* and *ndhD* genes, respectively. The *ndhB* gene was characterized by the highest number of potential editing sites (12 sites), followed by *ndhD* gene possessing 8 sites.

### 3.3. Comparative Analysis of Chloroplast Genome Structure

**Figure 3:**
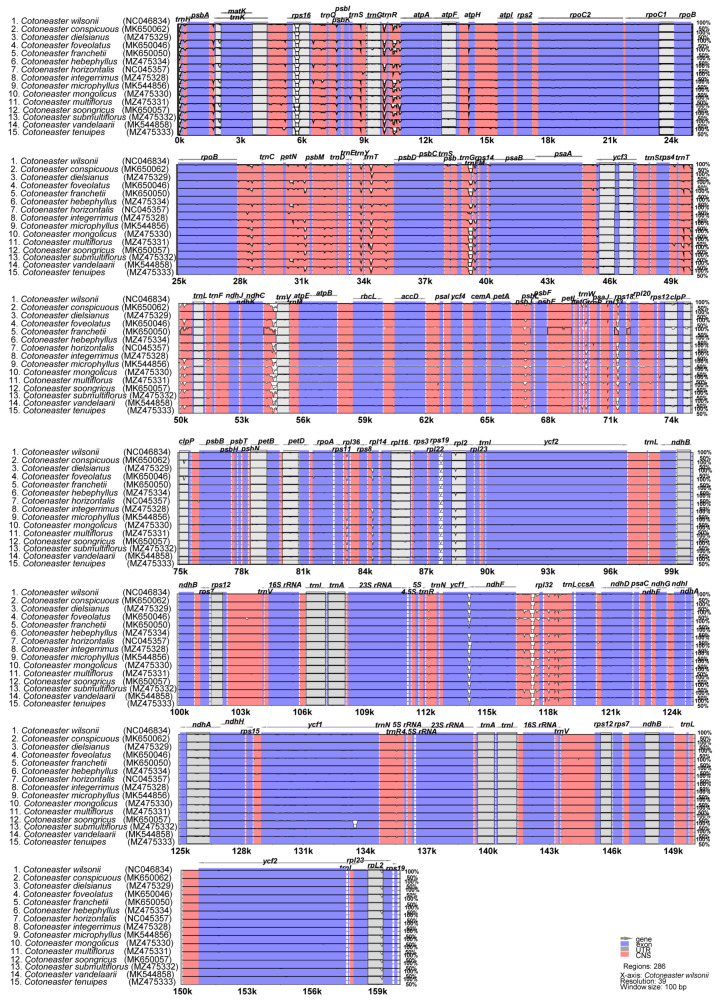
Visualization of alignment of the 15 plastome sequences of *Cotoneaster* species.

**Figure 4:**
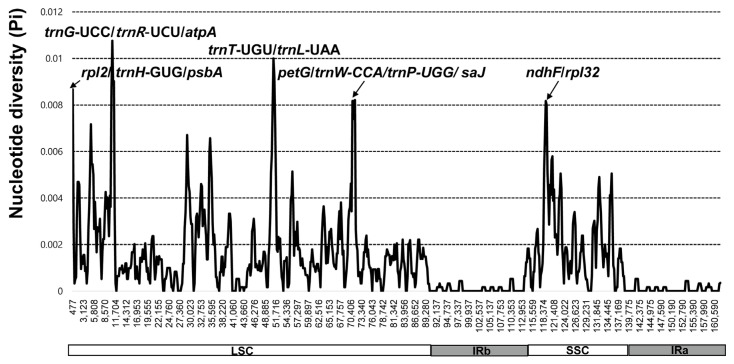
Sliding window analysis of the 15 whole-chloroplast genomes of *Cotoneaster* species.

The plastomes of 14 *Cotoneaster* species (i.e., *C. conspicuous*, *C. dielsianus*, *C. foveolatus*, *C. franchetii*, *C. hebephyllus*, *C. horozontalis*, *C. integerimus*, *C. microphyllus*, *C. mongolicus*, *C. multiflorus*, *C. soongricus*, *C. submultiflorus*, *C. vandelaarii*, and *C. tenuipes*) were plotted using mVISTA, using the annotated *C. wilsonii* plastome as a reference (Figure 3). The results indicated that the LSC region was the most divergent, whereas the two IR regions were highly conserved. Furthermore, the non-coding regions were found to be more divergent and variable than the coding regions. Sliding window analysis performed using the DnaSP program revealed highly variable regions in the plastomes of 15 *Cotoneaster* species (Figure 4). Comparison of these 15 plastomes revealed that the average value of nucleotide diversity (Pi) over the entire chloroplast genome was 0.001345, with the most variable region (Pi = 0.01076) being the *trnG*-UCC/*trnR*-UCU/*atpA* intergenic region. One additional intergenic region, *trnT*-UGU/*trnL*-UAA, was also highly variable (Pi = 0.01). Three additional variable regions with Pi values greater than 0.008 included *rpl2*/*trnH*-GUG/*psbA* (Pi = 0.00867), *petG*/*trnW*-CCA/*trnP*-UGG/*psaJ* (Pi = 0.00821), and *ndhF*/*rpl32* (Pi = 0.00817).

### 3.4. Identification of Genes under Positive Selection

Positive selection analysis allowed us to identify positively selected genes among the 15 *Cotoneaster* plastomes (Table 2). Among the conserved genes, eight genes with positively selected sites were identified with an effectively significant LRT *p*-value (Table 2). These genes included the c-type cytochrome synthesis gene (*ccsA*), maturase K gene (*matK*), three NADH dehydrogenase subunit genes (*ndhD, ndhF*, and *ndhK*), cytochrome f precursor gene (*petA*), Rubisco gene (*rbcL*), and mitochondrial ribosomal protein L16 gene (*rpl16*), and based on the M8 model, all eight genes had one positive site. However, most of the genes, i.e., 68 of 76 genes had an average Ka/Ks ratio below 1, indicating that these genes have been subjected to the strong purifying selection in the *Cotoneaster* chloroplast.

### 3.5. Phylogenetic Analysis

**Figure 5:**
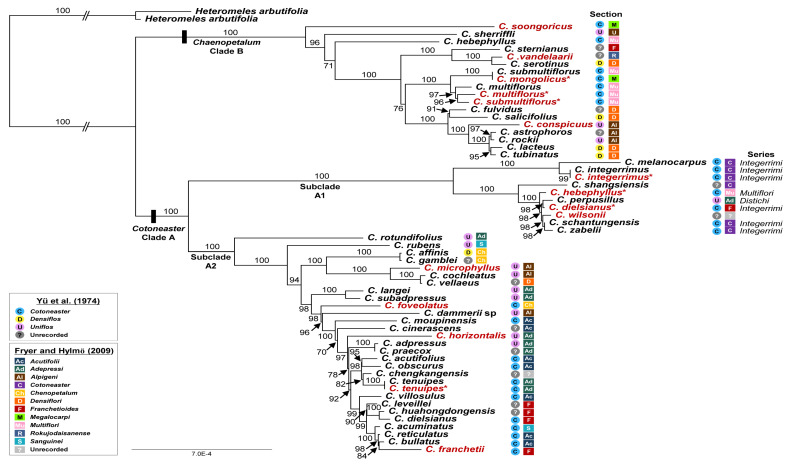
Maximum likelihood tree inferred from 57 species of *Cotoneaster* and two *Heteromeles* plastomes used as the outgroup. Bootstrap values based on 1000 replicates are shown on each node. Fifteen accessions included in the comparative analyses are shown in red and of these 15 accessions, seven newly sequenced accessions in the current study are indicated using asterisks [1,5].

Based on a total of 139,251 nucleotide sites and 1217 parsimony informative sites, maximum likelihood analysis conducted on the best-fit model of “TVM + F + R2” enabled us to infer phylogenetic relationships among 57 accessions of *Cotoneaster* plastomes, including *C. wilsonii* (Figure 5). The plastid phylogenomic tree, including seven newly sequenced species in this study, confirmed the earlier species relationships [4]. Two major lineages within the genus almost corresponding to two subgenera, *Cotoneaster* (Clade A) and *Chaenopetalum* (Clade B), were identified with strong support (100% bootstrap support, BS) (Figure 5). Within the clade of *Cotoneaster* (Clade A), two somewhat divergent lineages (subclade A1 and subclade A2) were found with strong BS support (100% each). Subclade A1 included several species of sect. *Cotoneaster* (*C. integerrimus*, *C. shangsiensis*, *C. schantungensis*, and *C. zabelii*), sect. *Multiflori* (*C. hebephyllus*), sect. *Adpressi* (*C. perpusillus*), and sect. *Franchetioides* (*C. dielsianus*) sensu Fryer and Hylmö [1] and Ulleung Island endemic *C. wilsonii*. All but three species (*C. hebephyllus*, *C. perpusillus*, and *C. dielsianus*) belonged to sect. *Cotoneaster* series: *Integerrimi* sensu Yü et al. [5]. Regarding the phylogenetic position of *C. wilsonii*, the plastome phylogenetic tree revealed that it shared its most recent common ancestor with *C. schantungensis* and *C. zabelii*, which are endemic to Shandong and central/northwestern China (including Shandong), respectively (97% BS).

As for the phylogenetic relationships of several conspecific plastomes between this study and Meng et al. [4], we found some congruences as well as incongruences. For example, *C. submultiflorus* (208-2000-A; originally from Gansu) sequenced in this study was closely related to *C. multiflorus* (1134-A; originally from Shaanxi) and *C. multiflorus* (Yunnan) [4]. *Cotoneaster submultiflorus* (Xinjiang) [4] was a sister to *C. mongolicus* (1007-86-A, originally from Guangdong). All these accessions formed a clade with 100% BS support in clade B. Although these accessions were part of the monophyletic group, other conspecific plastomes showed drastically different positions. For example, *C. hebephyllus* (sect. *Multiflori*) (Tibet) [4] represented an early diverged lineage within *Chaenopetalum* (Clade B), but the accession sampled in this study (68-85-A; Arnold Arboretum) was a sister to the clade containing *C. perpusillus*, *C. dielsianus*, *C. wilsonii*, *C. schantungensis*, and *C. zabelii* (100% BS). Furthermore, *C. dielsianus* (sect. *Franchetioides*) sampled in this study (Hubei; 13428-B, Arnold Arboretum) was closely related to *C. perpusillus* (sect. *Adpressi*; 98% BS) in subclade A1, whereas the other accession (Yunnan) [4] was a sister to *C. huahongdongensis* (sect. *Franchetioides*, 100% BS) in subclade A2. These two accessions, *C. dielsianus* (Yunnan) and *C. huahongdongensis*, were sampled from Yunnan, with a direct distance of <70 km (Supplementary Table S2 of Meng et al. [4]). Given the wide disjunct distribution of *C. dielsianus* in Sichuan and Hubei, it is uncertain whether *C. dielsianus* sampled from Hubei in this study represents a different taxon. We unwittingly sequenced *C. tenuipes* (sect. Adpressi; 7276-C, Arnold Arboretum), the same accession that was sequenced by Meng et al. [4], and found that they have identical sequences, ruling out the possibility of sequencing mistakes between the two studies.

## 4. Discussion

### 4.1. Chloroplast Genome Structure and Evolution in Genus *Cotoneaster*

In this study, we assembled and characterized seven additional species of *Cotoneaster* (*C. dielsianus*, *C. hebephyllus*, *C. integerrimus*, *C. mongolicus*, *C. multiflorus*, *C. submultiflorus*, and *C. tenuipes*) and added them to the existing chloroplast genome database of the genus [4]. Furthermore, for the first time, we performed several comparative analyses of plastomes based on seven newly sequenced and seven major lineages within the genus, including *C. wilsonii* on Ulleung Island, to gain an insight into plastome evolution. The complete chloroplast genome size in *Cotoneaster* ranged from 159,521 bp (*C. acutifolius*) to 160,016 bp (*C. hebephyllus*), and the largest plastome from Meng et al. [4] belonged to *C. melanocarpus* (159,970 bp). Thus, we have characterized and added the largest plastome found within genus *Cotoneaster* to date. There were differences of less than 500 bp in the complete length of the plastomes, indicating their conservation within *Cotoneaster*. Given the highly conserved nature of plastomes, no structural variation or gene content rearrangement was found within the genus (Table 1). As expected, the LSC region was the most divergent, whereas the two IR regions were highly conserved. Furthermore, non-coding regions were found to be more divergent and variable than the coding regions. These findings are consistent with the patterns observed in common angiosperms [22,27,29,30,39,58]. The GC content of the complete plastomes in the 15 representative *Cotoneaster* species was identical (36.6%), and this high GC content could be attributed to the high GC content in the IR regions [59].

As we compared the 15 representative plastomes of *Cotoneaster* in this study, we revealed the retention of an intron in *atpF* belonging to group II introns [60]. Intron loss or gain in the plastome can be an evolutionarily significant event as introns are highly conserved among land plants [61]. Loss of the *atpF* intron has been reported in several Rosaceae genera, such as *Fragaria*, *Rosa*, *Potentilla*, and *Rubus* [34,37,62]. These genera belong to subfamily Rosoideae, and thus it seems that the loss of introns within *atpF* genes has occurred once within this subfamily. In contrast, several other genera of Rosaceae belonging to subfamily Amygdaloideae, such as *Cotoneaster, Alchemilla, Malus*, *Prunus*, *Pyrus*, and *Sorbus*, retain introns within *atpF* genes [62,63]. As suggested, it is unclear whether intron loss has occurred in the species of subfamily Rosoideae, genera in the family Rosaceae, and families in the order Rosids, and whether this has phylogenetic significance, utility in the classification system, and forms a potentially resourceful evolutionary maker in angiosperms.

### 4.2. The Codon Usage Pattern in the Cotoneaster Chloroplast Genome

The frequency of codon usage in the 15 *Cotoneaster* plastomes was determined based on the sequences of protein-coding genes (Supplementary Table S2). The preferential use of codons during gene translation (i.e., specific codons are used more often than others) is known as codon usage bias, and codon usage values are described by the relative synonymous codon usage (RSCU) [43]. RSCU is the ratio between the expected frequency of use and the actual frequency usage of a particular codon. RSCU values less than 1 indicate lower frequency usage than expected, whereas values greater than 1 indicate a higher usage frequency [52]. The codon usage bias and any intraspecific or interspecific codon usage variation are indicative of selective constraints on codon choice. We found the highest RSCU value in the usage of the UUA codon for leucine (1.92–1.94) followed by that of GCU for alanine (1.83–1.84) and AGA for arginine (1.82–1.84), whereas the lowest value was found in the usage of AGC for serine (0.38–0.39) and GAC for aspartic acid (0.37–0.38) (Supplementary Table S2). Codons AUG (M) and UGG (W) encoding methionine and tryptophan showed no bias (RSCU = 1). This pattern is consistent with that of the genus *Malus*, belonging to the same subfamily Amygdaloideae [58]. Similar to other Rosaceae species (*Potentilla* L. and *Spiraea*, L. [62]; *Alchemilla* L., [63]; *Malus*, Cho et al., [58]), we found that codon usage was biased toward a high RSCU value for U and A at the third codon position in genus *Cotoneaster*.

### 4.3. The Characteristic of RNA Editing Sites in the *Cotoneaster* Chloroplast Genome

Although previous studies have demonstrated variable numbers of RNA editing sites among higher taxonomic ranks of land plants and between the two organellar genomes [64,65,66], the extent of variation among closely related species or multiple genera of the same family is known to be sporadic. In organellar genomes (chloroplast and mitochondria), conversion from C (cytidine) to U (uridine) has been shown to be the most prevalent [67,68]. Regarding the RNA editing sites in the 15 *Cotoneaster* plastomes, all species shared RNA editing sites in 14 photosynthesis-related genes, seven self-replication genes, and three other functional genes (Supplementary Table S3). All 15 *Cotoneaster* species also shared the same 10 genes without any RNA editing sites. Although RNA editing sites are highly conserved among closely related species, we found that *C. microphyllus*, belonging to *Cotoneaster* Clade A, subclade A2 (Figure 3), exceptionally, contains one additional gene, *rps8*: with RNA editing site conversion from ACC (T, threonine) to ATC (I, isoleucine). This is in contrast to the *Malus* plastomes from East Asia, which showed that the *rps8* gene did not have an RNA editing site [40]. Three other functional genes (*accD*, *clpP*, and *matK*) and seven self-replication genes contained RNA editing sites and were common between *Malus* and *Cotoneaster*; however, we found that the *petG* gene, reported to have an RNA editing site in *Malus,* did not have an RNA editing site in the *Cotoneaster* species surveyed in this study. As shown in previous studies [40,62,65,69], the highest number of potential editing sites were found in the NADH dehydrogenase genes, with the *ndhB* gene harboring 12 sites, and *ndhD* gene harboring 8 sites. Similar to *Malus*, we found that the highest conversions in the editing sites were represented by changes from serine (S) to leucine (L) (average confidence score of 23.81) followed by proline (P) to leucine (L) (average confidence score of 8.86).

As an important locus for phylogenetic analysis, the identification of hotspot regions or highly variable regions of the chloroplast genome is important to disentangle the complex evolutionary history of *Cotoneaster*, especially for dissecting reticulation and polyploidization [2,4,70]. We identified the hotspot regions based on the 15 *Cotoneaster* plastomes, including *trnG*-UCC/*trnR*-UCU/*atpA* (Pi = 0.01076), *trnT*-UGU/*trnL*-UAA (Pi = 0.01), *rpl2*/*trnH*-GUG/*psbA* (Pi = 0.00867), *petG*/*trnW*-CCA/*trnP*-UGG/*psaJ* (Pi = 0.00821), and *ndhF*/*rpl32* (Pi = 0.00817). Of these, the same region was identified as a hotspot by comparison with other Rosaceae genera, such as *trnT*/*trnL* (*Rubus*, [34]), *trnH*/*psbA* (*Alchemilla*, [63]; *Amygdalus*, [71]), *trnR*/*atpA* (*Rosa*, [72]; *Fragaria*, [73]), and *ndhF*/*rpl32* (*Rosa*, [72]; *Alchemilla*, [63]). Therefore, these hotspots and other highly variable regions of family Rosaceae [30,74,75,76] could be useful in population genetics and phylogenetic studies.

### 4.4. Positively Selected Genes in Cotoneaster Chloroplast Genomes

Most plastome genes have evolved under purifying selection because of functional limitations throughout chloroplast genome evolution [76,77,78,79]. As synonymous nucleotide substitutions occur more frequently than non-synonymous substitutions, Ka/Ks values are usually less than 1 [80]. In the 15 plastomes of genus *Cotoneaster*, most of the genes have been under strong purifying selection; 68 of the 76 genes have an average Ka/Ks ratio below 1. However, among the 15 representative species selected in *Cotoneaster*, eight genes, that is, *ccsA*, *matK*, *ndhD*, *ndhF*, *ndhK*, *petA*, *rbcL*, and *rpl16*, have undergone selective pressure. Positive selection of several functional genes has been previously reported in several studies. For example, the *rbcL* gene, which encodes the large subunit of RuBisCO, plays an important role in photosynthesis and is often under positive selection in various plant groups including *Fragaria* [76], *Gossypium* L. [81], *Panax* L. [78], *Paulownia* [79], Poaceae grass after the C3-C4 photosynthetic transition [82], and *Rubus* [34]. Based on the current analysis, it is also likely that the *rbcL* gene was the target of selection during the evolution of *Cotoneaster*. The *matK* gene was identified to be under positive selection in *Cotoneaster*. The *matK* gene has also been shown to be under positive selection in several lineages within *Allium* L., suggesting its role in adaptation to a wide range of environments [36]. Positive selection of the *matK* gene has been reported in various other plant lineages, such as PACMAD grasses (Poaceae, [82]), *Chrysosplenium* L. [83], *Symplocarpus* Salisb. ex W.P.C.Barton [69], and *Rubus* [34]. As shown in several other plant groups (e.g., [36,84]), our study also revealed that three genes from the *ndh* family, that is, *ndhD*, *ndhF*, and *ndhK*, were under positive selection. Of the *ndh* gene family, *ndhK* has been shown to be positively selected in species adapted to different altitudinal habitats [25] and in shade-tolerant and sun-loving plants [85]. In addition, *ndhF* evolved under positive selection because of its involvement in the adaptation to hot and dry climates [86]. Therefore, these *ndh* gene family members likely contributed to adaptation to high light intensity during the evolution of *Cotoneaster*. It is also likely that the ribosomal protein-coding gene, *rpl16*, was selected to maintain the integrity of the protein synthesis machinery under various environmental stresses [87]. Overall, we hypothesized that these positively selected genes in different categories of the chloroplast genome, including subunits of cytochrome (*ccsA* and *petA*), are results of their important adaptive roles in diverse environmental conditions during the evolutionary radiation of the genus from the late Miocene to today.

### 4.5. Phylogenetic Position of Cotoneaster wilsonii on Ulleung Island

The origin and evolution of *C. wilsonii* on Ulleung Island have been problematic given their unusual geographic distribution in Korea. *Cotoneaster wilsonii*, which occurs very narrowly on Ulleung Island as a critically endangered species, represents the easternmost range of the entire genus *Cotoneaster*. Without natural distribution in the Japanese archipelago, only one additional species of *Cotoneaster*, *C. integerrimus*, is known to occur in North Korea. This species is also known to occur in a few isolated limestone areas in Gangwondo Province of South Korea (Samcheok city, Yeongwol-gun, and Jeongseon-gun), but its species identity and relationship with *C. wilsonii* are yet to be determined. Thus, considering its narrow geographic distribution in the oceanic Ulleung Island, which was formed approximately 1.8 million years ago, and the lack of a broad phylogenetic framework of the genus, the origin and phylogenetic relationships of *C. wilsonii* relative with other congeneric species has been a matter of speculation. With the broad scale phylogenomic study by Meng et al. [4] and our current study, we, for the first time, assessed the phylogenetic position of *C. wilsonii*. Although several cases of incongruences between nuclear and chloroplast phylogeny in *Cotoneaster* caused by hybridization and incomplete lineage sorting were revealed, a tentative conclusion about the phylogenetic position of *C. wilsonii* can be suggested based on the congruence in the clade of our interest (Figure 4 of Meng et al. [4]).

Based on morphological similarity and shared flavonoid profiles, Chang and Jeon [14] suggested that *C. multilforus* would be the closest continental sister species of *C. wilsonii*, or that they are conspecific. *Cotoneaster multiflorus* and *C. hebephyllus* contain flavone O-glycosides identical to those in *C. wilsonii*, suggesting their possible role in the origin of *C. wilsonii* on Ulleung Island. *Cotoneaster multiflorus* and several related species belong to sect. *Cotoneaster* series *Multiflori* sensu Yü et al. [5]. Our current study strongly suggests that *C. wilsonii* shares its most recent common ancestor with two species, *C. schantungensis* and *C. zabelii*, which belong to sect. *Cotoneaster* series *Integerrimi* sensu Yü et al. [5] (Figure 5). Unlike *C. multiflorus*, which has 5–21 flowers per inflorescence and spreading petals, the two most closely related species, *C. schantungenis* and *C. zabelii*, tend to have fewer flowers, 3–6 or 3–10 (or more), respectively [7]. In addition, both *C. zabelii* and *C. schantungensis* have erect petals, whereas *C. wilsonii* has 4–17 flowers (average of 10 flowers per corymb) and spreading petals. While the clade containing *C. wilsonii* belongs to *Cotoneaster* (Clade A), *C. multiflorus* and related species all belong to sect. *Cotoneaster* series *Multiflori*, which belongs to a different clade, i.e., *Chaenopetalum* (Clade B). Therefore, it is less likely that *C. multiflorus* and related species are involved in the origin of *C. wilsonii*, implying that their morphological similarities and similar flavonoid profiles are most likely convergent features or symplesiomorphy. Furthermore, the phylogeny of 203 low-copy nuclear genes also suggested that *C. multiflorus* and *C. hebephyllus* are not closely related to the clade containing *C. wilsonii* [4], thus corroborating our current results. Furthermore, the chromosome number of *C. wilsonii* is known to be diploid (2n = 34) [88], whereas *C. multiflorus* is tetraploid (2n = 68) [7]. The accession of *C. hebephyllus* (68-85-A) from the Arnold Arboretum with its wild origin unknown is related to the *C. wilsonii*-containing clade, but the wild origin accession (Tibet) by Meng et al. [4] is distantly related to *C. wilsonii*. In fact, the *C. hebephyllus* accession from Tibet is a sister to the clade containing the species of sect. *Multiflori* and other sections sensu Fryer and Hylmö [1]. Therefore, we are uncertain about the species identity of the *C. hebephyllus* accession at Arnold Arboretum. Taking this caveat together with the current phylogeny obtained, we can safely rule out the possibility of sect. *Cotoneaster* series *Multiflori* sensu Yü et al. [5] being involved in the origin of *C. wilsonii*. Rather, it is highly likely that the species in sect. *Cotoneaster* series *Integerrimi* was involved in the origin of *C. wilsonii*. The chloroplast phylogenomic tree suggests that the sect. *Cotoneaster* series *Integerrimi* sensu Yü et al. [5] is not monophyletic (Figure 5). Of several species from series *Integerrimi*, it seems likely that a common ancestor shared with species such as *C. schantungensis* and *C. zabelii* was involved in the origin of *C. wilsonii* on Ulleung Island. *Cotoneaster schantungensis* is endemic to Shandong Province, which is geographically close to the Korean Peninsula, just across the Yellow Sea. Furthermore, *C. zabelii* occurs quite broadly in western Qinghai, northeastern Nei Mongol, and eastern Shandong [7].

As part of the clade containing *C. wilsonii*, the potential involvement of *C. dielsianus* in the origin of Ulleung Island is also plausible. *C. dielsianus* belongs to the same sect. *Cotoneaster* series *Integerrimi* sensu Yü et al. [5] or sect. *Franchetioides* sensu Fryer and Hylmö [1] but is morphologically distinct from *C. wilsonii* by having 3–7 small (6–7 mm in diameter) flowers, abaxially villous hypanthium, erect petals, and 3 (rarely 5) styles [7]. In contrast, *C. wilsonii* has 4–17 (average of 10) large (8–12 mm) flowers, an abaxially glabrous hypanthium, spreading petals, and 2 (rarely 3) styles. The accession (13428-B) of *C. dielsianus* from the Arnold Arboretum, which was originally collected from western Hubei, contained a very different plastome compared to the one sequenced by Meng et al. [4], which was sampled from Yunnan. *Cotoneaster dielsianus* occurs somewhat broadly, ranging from central to southwestern China, and without examining the voucher specimen of the Yunnan accession, it is difficult to determine whether these two accessions represent distinct taxa or infraspecific variation within *C. dielsianus*. The highly polyphyletic sect. *Cotoneaster* series *Integerrimi* sensu Yü et al. [5] or sect. *Francheotioides* sensu Fryer and Hylmö [1] in the *Cotoneaster* phylogeny further complicate the resolution of this issue.

The plastome phylogenetic position of *C. perpusillus* as part of a clade containing *C. wilsonii* and related species (subclade A1) seems unusual given its morphology and sectional/serial assignment. *Cotoneaster perpusillus* belongs to the sect. *Uniflos* sensu Yü et al. [5] and sect. *Adpressi* sensu Fryer and Hylmö [1]. It is currently recognized as *C. horizontalis* var. *perpusillus*, known to occur in central China (Guizhou, Hubei, Shaanxi, and Sichuan), with characteristics of having only one or two flowers, smaller leaves (<1 cm), and erect petals [7]. With the exclusion of *C. hebephyllus*, this is the only species of sect. *Adpressi* or *Uniflos*, placed in the clade of sect. *Cotoneaster* sensu Fryer and Hylmö [1] and sect. *Cotoneaster* series *Integerrimi* sensu Yü et al. [5]. The accession of *C. perpusillus* sequenced by Meng et al. [4] was collected from Yunnan, which is a neighboring Sichuan Province. Although it is a part of subclade A1, *C. perpusillus* is a sister to *C. harrysmithii* (albeit weakly supported, with a Bayesian posterior probability of 0.88, and Ultrafast bootstrap support value < 50%) in the species tree based on 203 low-copy nuclear genes [4]. *Cotoneaster harrysmithii*, which occurs rather narrowly in western Sichuan and southeastern Xizang, belongs to sect. *Uniflors* sensu Yü et al. [5], and sect. *Adpressi* sensu Fryer and Hylmö [1], which is the same sectional assignment as *C. perpusillus*. Thus, it is highly likely that *C. perpusillus* experienced hybridization events with *C. dielsianus*, which also occurs in central and southwestern provinces (including Yunnan and Sichuan), and subsequently captured the chloroplast of *C. dielsianus*, its sister species in the chloroplast phylogenomic tree.

Based on the broad phylogenomic framework and molecular dating of *Cotoneaster*, we can also gain an insight into the timing of *C. wilsonii* on Ulleung Island, Korea. Meng et al. [4] suggested that the crown node age for the subclade A1, including *C. wilsonii* and related species, is estimated to be 6.25 million years (MY) old. In addition, the clade containing *C. perpusillus*, *C. schantungensis*, and *C. zabelii*, is estimated to be 0.72 MY, whereas the clade containing all these species plus *C. schangsiensis* is 2.41 MY old. As *C. wilsonii* is sister to the clade of *C. schantungensis* and *C. zabelii*, the most recent common ancestor of *C. wilsonii* and *C. schantungensis*, *C. zabelii*, should be younger than 0.72 MY. This suggests that *C. wilsonii* may have originated very recently, long after the formation of Ulleung Island, which is slightly less than 2 MY old. Although nearly 40 vascular endemic species occur on Ulleung Island, little is known about their timing of origin. Thus, further investigation based on a robust and well-resolved phylogenetic framework and molecular dating is required to better understand the temporal scale of these endemic assemblages on the island.

## Figures and Tables

**Table 1:** Summary of the characteristics of the seven *Cotoneaster* chloroplast genomes analyzed in this study.

Taxa	*Cotoneaster dielsianus*	*Cotoneaster hebephyllus*	*Cotoneaster integerrimus*	*Cotoneaster mongolicus*	*Cotoneaster multiflorus*	*Cotoneaster submultiflorus*	*Cotoneaster tenuipes*
Total cpDNA size (bp)	159,898	160,016	159,996	159,797	159,855	159,614	159,595
GC content (%)	36.6	36.6	36.6	36.6	36.6	36.6	36.6
LSC size (bp)/ GC content (%)	87,901/34.3	87,901/34.3	87,813/34.3	87,818/34.3	87,903/34.3	87,786/34.3	87,592/34.3
IR size (bp)/ GC content (%)	26,397/42.6	26,397/42.6	26,501/42.6	26,371/42.7	26,371/42.7	26,371/42.7	26,382/42.7
SSC size (bp)/ GC content (%)	19,203/30.5	19,203/30.4	19,181/30.3	19,237/30.5	19,210/30.5	19,086/30.5	19,239/30.4
Number of genes	131	131	131	131	131	131	131
Number of protein-coding genes	84	84	84	84	84	84	84
Number of tRNA genes	37	37	37	37	37	37	37
Number of rRNA genes	8	8	8	8	8	8	8
Number of duplicated genes	17	17	17	17	17	17	17
Accession number	MZ475329	MZ475334	MZ475328	MZ475330	MZ475331	MZ475332	MZ475333
Total reads	50,739,498	49,015,178	49,728,586	52,448,006	54,743,088	42,975,682	51,539,694
Coverage of sequences	721	149	223	582	229	160	755

**Table 2:** Log-Likelihood values of site-specific models, with detected sites having dN/dS values > 1.

Gene Name	Models	np	ln L	Model Compared	Likelihood Ratio Test *p*-Value	Positively Selected Sites
*ccsA*	M8	33	−1330.734734	M7 vs. M8	0.0000	75 L 0.962 *
	M7	31	−1379.715271			
*matK*	M8	33	−2086.970879	M7 vs. M8	0.0000	405 V 0.990 *
	M7	31	−2134.673613			
*ndhD*	M8	33	−2119.842957	M7 vs. M8	0.0000	32 T 0.995 **
	M7	31	−2177.429276			
*ndhF*	M8	33	−3048.774585	M7 vs. M8	0.0011	489 I 0.989 *
	M7	31	−3055.570081			
*ndhK*	M8	33	−1282.922705	M7 vs. M8	0.0000	11 T 0.953 *
	M7	31	−1809.996500			
*petA*	M8	33	−1349.032847	M7 vs. M8	0.0000	154 S 0.959 *
	M7	31	−1401.590518			
*rbcL*	M8	33	−1970.087905	M7 vs. M8	0.0000	255 I 0.969 *
	M7	31	−2026.722719			
*rpl16*	M8	33	−657.974758	M7 vs. M8	0.0000	4 P 0.998 **
	M7	31	−652.786169			

* *p* < 0.05; ** *p* < 0.01, np represents the degree of freedom.

## Data Availability

The datasets generated and/or analyzed during this study can be found in GenBank, National Center for Biotechnology Information (http://www.ncbi.nlm.nih.gov/genbank/, (8 January 2022) under accession numbers MZ475328 and MZ475334.

## Supplementary Materials

The following supporting information can be downloaded at: https://www.mdpi.com/article/10.3390/genes13050728/s1, Table S1. List of seven *Cotoneaster* species obtained from Arnold Arboretum, Harvard University. NA = not available; Table S2. The codon usage and codon-anticodon recognition pattern for RNA in the 15 *Cotoneaster* plastomes; Table S3. Predicted RNA editing sites in the complete chloroplast genomes of the 15 *Cotoneaster* species.
